# Health intervention trials involving transgender, transabled and transracial persons in Africa: A scoping review

**DOI:** 10.1002/puh2.182

**Published:** 2024-05-06

**Authors:** Jimoh Amzat, Kehinde Kazeem Kanmodi, Kafayat Aminu, Abbas Ismail, Afeez Abolarinwa Salami

**Affiliations:** ^1^ Department of Sociology Usmanu Danfodiyo University Sokoto Nigeria; ^2^ School of Health and Life Sciences Teesside University Middlesbrough UK; ^3^ Faculty of Dentistry University of Puthisastra Phnom Penh Cambodia; ^4^ School of Dentistry University of Rwanda Kigali Rwanda; ^5^ Cephas Health Research Initiative Inc Ibadan Nigeria; ^6^ Center for Child and Adolescent Mental Health University College Hospital Ibadan Nigeria; ^7^ Department of Sociology Umaru Musa Yar'adua University Katsina Nigeria; ^8^ Department of Oral and Maxillofacial Surgery University College Hospital Ibadan Nigeria

**Keywords:** Africa, health trial, interventions, scoping review, trans persons, transabled, transgender, transracial

## Abstract

**Background:**

Health intervention trials constitute important research efforts to find appropriate solutions to health issues affecting different populations. In many cases, it involves high‐risk groups such as the trans‐communities. This scoping review aims to review the existing health intervention trials involving transgender, transabled and transracial persons in Africa.

**Methods:**

This scoping review adopted the research design by Arskey and O'Malley. Using the Population–Concept–Context framework, a robust systematic search of four research databases, including APA PsycINFO, SCOPUS, CINAHL Complete and PubMed, was conducted to retrieve literature relevant to the review's question. Duplicate copies in the retrieved literature were removed using the Rayyan web‐based application. The residual literature was screened for relevance based on the review's inclusion and exclusion criteria, and only those eligible articles were included in this review. From the included literature, data were charted, collated, summarized and presented as results.

**Results:**

The scoping review included and reviewed only four articles, which reported studies involving transgender persons. No peer‐reviewed original research article on transabled and transracial persons in Africa was found eligible for inclusion in this review. All the reviewed articles focused on at‐risk, healthy and human immunodeficiency virus (HIV)‐uninfected adult participants ranging between the ages of 18 and 65 years. The domains investigated in those articles were on sexual health, HIV preventive drugs and vaccine trials. The reviewed findings showed the use of HIV‐inhibiting medications and HIV screening or testing as vital preventive interventions among transgender persons in Africa. The available research evidence shows sexuality reductionism about trans behaviour by neglecting other health domains.

**Conclusion:**

Health trial research on transracial, transgender and transabled persons is a largely underexplored research domain in Africa. More health intervention trials, beyond the domain of sexual health, are required to improve the health and well‐being of this highly marginalized population group in Africa.

## Graphical abstract

Four articles were included in this scoping review.

All the included articles were on intervention trial studies involving transgender persons in Africa; however, none of them investigated transabled and transracial persons.

The four articles were about sexual health, HIV preventive drugs and vaccine trials involving transgenders.

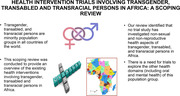

## INTRODUCTION

Transgender, transabled and transracial individuals constitute minority populations worldwide [1, 2]. A transgender person is someone whose current gender identity differs from the sex assigned at birth [3]. A transabled individual undergoes surgical amputation of a healthy organ due to a condition known as body integrity identity disorder [4]. Transracial individuals identify with a racial or ethnic group different from their birth race. Across Africa, research output on trans individuals remains notably low compared to the general population, with limited data available on key health indicators, such as morbidity, mortality and life expectancy [5, 6]. Unfortunately, trans individuals bear a disproportionately high burden of diseases due to stigma, criminalization and social inequalities [7, 8].

There is glaring inadequate data concerning the significant public health challenges faced by trans individuals in Africa. Consequently, there is an urgent need for more research within trans communities to comprehend their health issues and develop effective interventions. Health research, particularly intervention trials, plays a crucial role in devising tailored, evidence‐based solutions to address the health disparities experienced by trans communities [9, 10].

Although numerous health intervention trials have been conducted across various population groups in Africa [11, 12], no comprehensive scoping review has been undertaken to assess the evidence and gaps in health trials involving transgender, transabled and transracial populations. The findings of such a review would enlighten both the global and African scientific and public communities about the current status of research in this area, thereby guiding future research endeavours and public health initiatives aimed at improving the health and well‐being of these marginalized groups in Africa. Consequently, this scoping review was undertaken to provide an overview of existing health interventions involving transgender, transabled and transracial individuals in Africa.

## METHODS

### Design

The scoping review design adopted for this study was the one prescribed by Arskey and O'Malley [13]. Moreover, the review was reported based on the Preferred Reporting Items for Systematic Reviews and Meta‐Analyses extension for Scoping Reviews’ (PRISMA‐ScR) checklist for documenting scoping reviews [14].

### Literature identification

The Population–Concept–Context framework guided the literature search strategy used for this scoping review. The populations of interest were transgender, transabled and transracial persons; the concept was a health intervention trial, and the context was Africa.

Four research databases, including APA PsycINFO, SCOPUS, CINAHL Complete and PubMed, were systematically searched on 21 January 2023 to retrieve literature relevant to the scoping review using a combination of search terms and with the aid of Boolean operators (‘OR’ and ‘AND’) (see the search strings in Tables S1–S3). The search did not adopt the use of time limiters; all literature published from inception till 21 January 2023 was retrieved from the search. The search was conducted by two authors (KKK and AAS).

### Selection of literature

Duplicate copies in the retrieved literature were removed using the Rayyan web‐based application. After deduplication, the remaining literature was screened for inclusion into (or exclusion from) the review by two independent reviewers, based on the scoping review's selection criteria. The screening process was two‐staged. Literature title and abstract screening were done in the first stage (by KKK and AAS), whereas full‐text screening was done in the second stage (by KKK and KA) (Table S4).

The inclusion criteria for this scoping review were peer‐reviewed original research articles which adopted a randomized trial (RT) design; articles published in English; articles reporting health interventions on transgender, transabled and transracial persons in Africa; and articles, the full texts of which are accessible. However, the criteria for excluding literature were: non‐original research articles published in peer‐reviewed journals (systematic reviews, scoping reviews, bibliometric reviews, editorials, notes, comments and letters); original research articles which did not adopt a randomized control trial design, including surveys, before and after studies, case–control studies, cohort studies and qualitative studies; articles that are not published in English; articles reporting health interventions on transgender, transabled and transracial persons in non‐African countries; articles reporting health interventions on non‐transgender, non‐transabled and non‐transracial persons in Africa; and articles, the full texts of which are accessible.

### Data charting, collation and summarization

The following data were charted from the included literature: names of authors; publication year; type of RT design, objectives/aims of the RT, study population characteristics, sample size, geographical location of the study and relevant findings answering the review's question. The charted data were collated, summarized and presented.

## RESULTS

**FIGURE 1:**
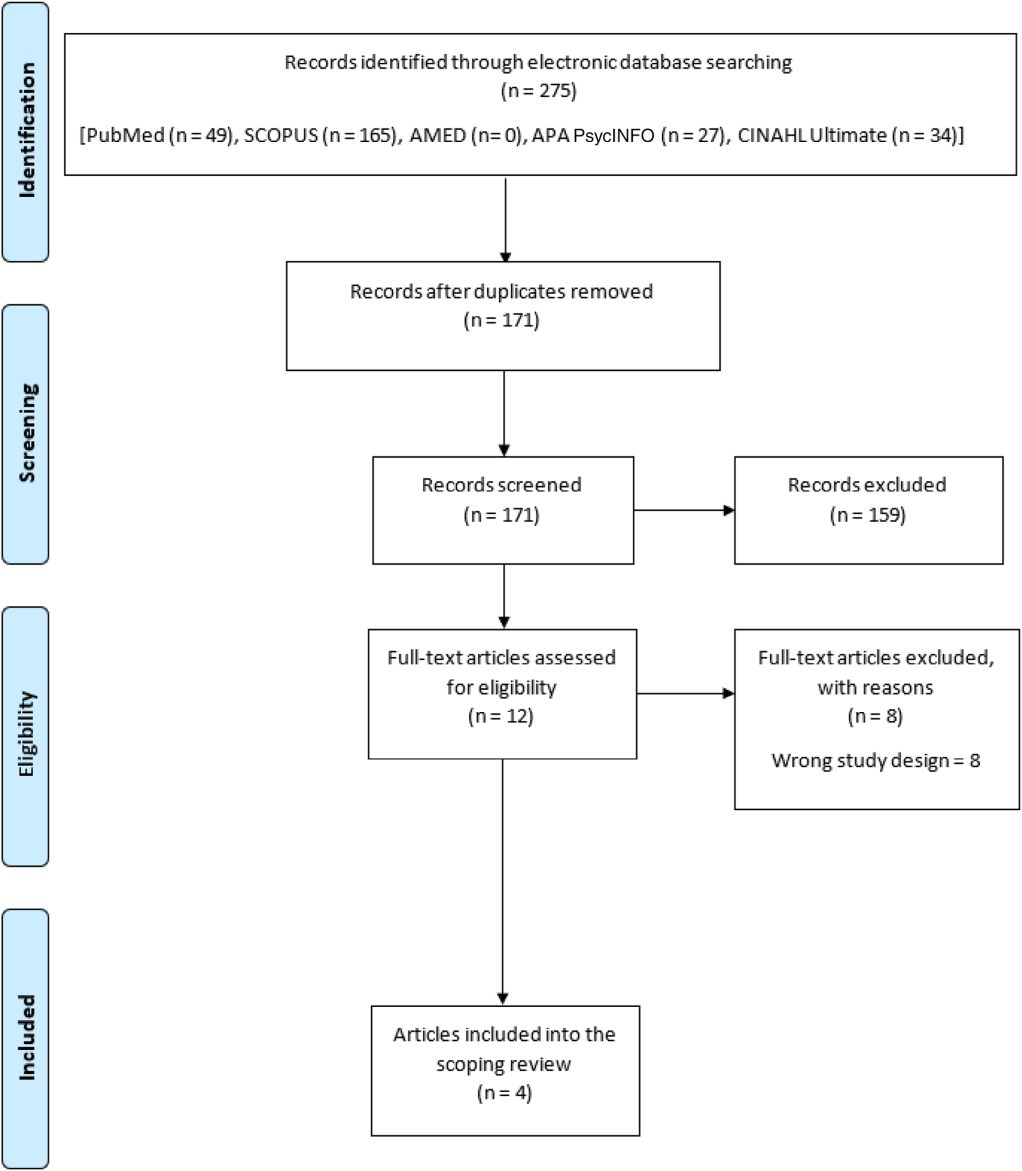
**Flow chart diagram**.

A total of 275 articles were retrieved from the database search. After deduplication, 104 papers were excluded. From the remaining 171 papers, only 4 were found to be relevant and, hence, included in the review (Table S4; Figure 1). Table 1 shows the characteristics of the included study, whereas Table 2 shows the summary of the articles included in this review.

**TABLE 1:** Characteristics of the included study.

Parameters	Frequency
**Population type**	
Transgender	4
Transabled	0
Transracial	0
**Age group**	
Children (<18 years)	0
Adults (≥18 years)	4
**Geographical location**	
North Africa	0
West Africa	0
Central Africa	0
East Africa	1
Southern Africa	3
**Investigated health domain**	
Oral health	0
Sexual and reproductive health	4
Mental health	0
Other domains of health	0
**Intervention trial type**	
Treatment trial	0
Prevention trial	4
**Trial design**	
Control trial design	2
Open‐label trial design	2
Study Setting	
Clinic‐based	1
Community‐based	3

**TABLE 2:** Health intervention trials involving transgender, transabled and transracial persons in Africa (Search date: 21 January 2023).

No.	Author and date	Sample size	Location/Country	Objectives	Study design and data source	Population	Intervention	Conclusion
1.	Cranston et al. (2017) [15]	195	Eight study locations were used: four in the United States (Boston, Pittsburgh, San Francisco and San Juan), two in Thailand (Bangkok and Chiang Mai), one in Peru (Lima) and one in South Africa (Cape Town)	To compare the safety profiles of daily oral antiretroviral combination emtricitabine/tenofovir disoproxil fumarate (FTC/TDF) tablet, daily rectal tenofovir (TFV) reduced‐glycerine (RG) 1% gel and receptive anal intercourse (RAI)‐associated rectal gel (TFV RG 1%). Moreover, to compare and estimate their acceptability as probable human immunodeficiency virus (HIV) prevention methods	A phase 2, three‐period, randomized sequence, open‐label, expanded safety and acceptability crossover study (Primary data)	Men who have sex with men (MSM) and transgender women (TGW)	Clinical trials	In TGW and MSM, administration of RG TFV gel via the rectum proved harmless. The likelihood of product use and adherence were comparable for the daily oral FTC/TDF regimen and intermittent gel regimen, but lower for the daily gel regimen
2.	Landovitz et al. (2018) [16]	In Cohort 1, 110 participants and in Cohort 2, 89 participants were eligible. A total of 200 participants were enrolled	Brazil, Malawi, South Africa and the United States	Evaluated the safety, tolerability and pharmacokinetics of long‐acting cabotegravir (CAB LA) among males and females who had not acquired HIV	Randomized, double‐blind, placebo‐controlled phase 2a trial. (Primary data)	HIV‐uninfected males and females aged 18–65 years. The sample population comprises transgender men (6) and TGW (1)	Trials	Examination at the recommended dosing intervals and doses revealed that CAB LA was well tolerated. Although ISRs were observed, they seldom resulted in product withdrawal. For all participants in the trial, the dosage of CAB LA 600 mg every 8 weeks satisfied the study's pharmacokinetic goals. The observed pharmacokinetic and safety data justify additional improvement of CAB LA, and research on the efficacy of CAB LA for the prevention and treatment of HIV is currently underway
3.	Corey et al. (2021) [17]	2699 participants were enrolled in HIV Vaccine Trials Network (HVTN) 704/HIV Prevention Trials Network (HPTN) 085, and 1924 participants were enrolled in HVTN 703/HPTN 081. Populations used in the primary analyses totalled 2687 and 1924, respectively	HVTN 704/HPTN 085, performed throughout Europe, South America and North America (the United States, Peru, Brazil and Switzerland). In sub‐Saharan Africa (South Africa, Zimbabwe, Malawi, Botswana, Kenya, Mozambique and Tanzania), HVTN 703/HPTN 081 was carried out	Designed to evaluate whether long‐term administration of a broadly neutralizing antibody (bnAb /VRC01) could prevent HIV‐1 acquisition and whether the susceptibility of the circulating viruses in the community to the bnAb would influence the efficacy of prevention in relation to gender or subtype. Moreover, whether it is possible to establish the in vitro level of VRC01 neutralization sensitivity of viruses as a biomarker of protection	Two parallel phase 2b, multicentre, randomized, double‐blind, placebo‐controlled, proof‐of‐concept efficacy trials (Primary data)	At‐risk cisgender men and transgender persons in Europe as well as the Americas. Sub‐Saharan African women who are believed to be at high risk	Trials	Although investigations of HIV‐1 isolates that are VRC01‐sensitive demonstrated with evidence that prophylaxis of antibodies that are broadly neutralizing (bnAb) may succeed, there was no significant difference between placebo and VRC01 in preventing global HIV‐1 infection
4.	Mujugira et al. (2022) [18]	117 enrolled 110 analysed (final study sample)	Kampala, Uganda	To test whether the combination of pre‐exposure prophylaxis (PrEP) and self‐testing for HIV (HIVST) influenced the acceptance and the utilization of the preventive interventions	Open‐label randomized Trial (Primary data)	Sex workers: female sex workers (FSWs) (84), TGW (14), MSM (10) and transgender men (2)	Trial	The randomized experiment revealed that HIVST did not aid adherence to PrEP among Ugandan sex workers. HIVST uptake was high. Utilization of the self‐testing tool as well as testing with sexual partners increased. Compliance with PrEP was low and believed to offer no protection against HIV

### Population type and age group

All reviewed articles focused on at‐risk, healthy, human immunodeficiency virus (HIV)‐uninfected population groups. However, in one of the articles, some participants were exposed to HIV subtype C and some to subtype B variants [17]. The groups were highly gender‐diverse and included transgender men and women [15, 16, 17, 18] as well as other groups, including sex workers [18], men having sex with men [15, 18] and cisgender women [17, 18].

An estimated 5128 people were recruited by the authors, including 197 transgender men and women [15, 16, 17, 18]. Three of the four articles sampled just a few numbers of transgender participants in their research. The sample of transgender in the studies was 16 (14 transgender women and 2 transgender men) [18], 19 (transgender women – 19) [15] and 7 (6 transgender men and 1 transgender woman) [16], respectively. Only one study sampled 155 transgenders (transgender women – 136 and transgender men – 19) in total [17].

All studies involved adult participants. Their ages ranged between 18 and 65 years [15, 16, 17, 18]. The lowest age range (18 to >35 years) was reported in the Ugandan study [18]. The highest median age was 31.1 years, whereas the lowest was 23 years.

Only one of the four studies was conducted within the community, as participants were interviewed and followed up in their various homes [18]. The remaining three investigations were completed within the clinic setting [15, 16, 17].

Only two of the reviewed articles documented baseline information. As indicated, most participants had an awareness of pre‐exposure prophylaxis (PrEP) (86.4%), were concerned about contracting HIV (80.0%), believed in the efficacy of PrEP (79.1%) and were concerned about PrEP side effects (60.0%) [18]. Furthermore, in another study, drug acceptability by the majority was also recorded at baseline [15].

All reviewed articles investigated issues related to sexual and reproductive health and specifically explored HIV prevention through diverse strategies.

### Intervention trial design

The four studies introduced HIV prevention trials in their research, such as self‐testing, clinic/laboratory testing, PrEP [18], HIV testing and vaccination [17], rectal and oral medications [15] as well as oral drugs and injections [16]. The difference between the two studies that introduced HIV testing [17, 18] as part of their intervention is the use of self‐testing in one [18] and clinical/laboratory testing in the other [17].

Two of the four papers adopted an open‐label RT research design [15, 18], and the other two utilized a double‐blind, placebo‐controlled trial [16, 17].

### Health intervention type

The reviewed articles introduced a combination of different health interventions to their study population. As earlier indicated, these health interventions were meant to prevent the spread of HIV. Some of the research utilized a variety of HIV prevention interventions: HIV self‐testing (HIVST), in‐clinic HIV screening and PrEP [17, 18]. In addition to oral and rectal medications for HIV prevention, counselling on HIV prevention and the diagnosis of sexually transmitted infection were offered in one of the cross‐site studies [15]. Another study offered oral lead‐in of cabotegravir (CAB) and injections of CAB or placebo to patients who had tested negative for HIV [16]. Lastly, some studies introduced HIV screening, counselling and PrEP as well as vaccination [17].

### Intervention outcomes

#### Adherence to and acceptability of health intervention

In Uganda, the introduced health intervention increased the rate of HIVST. HIVST acceptability and uptake by participants and their partners were high [18]. Another study also observed a high rate of adherence to HIV testing (94%). At the onset of their investigation, the uptake of infusion was likewise high [17]. However, compliance with daily oral PrEP remained significantly poor post‐intervention [18]. It was indicated that, irrespective of the assessment method used, the intervention did not improve PrEP uptake in the study groups. Hence, the uptake was significantly poor and failed to yield any protection against HIV [18].

Similarly, the preference for oral medication was significantly higher than daily gel use. Personal preference, in addition to ease of use and awareness about the efficacy of the regimen, determined the rate of adherence to each of the intervention drugs. Adherence to daily rectal gel was comparatively lower (70%) than receptive anal intercourse (RAI) rectal (80%) and daily oral regimens (90%) [15]. Another study also reported disinterest in HIV prevention and the non‐acceptability of oral PrEP among Africans, even when participants were counselled about drug availability and when drugs were provided free to participants [17]. However, compared to other groups, the preventive health intervention introduced had positive effects on PrEP adherence among some transgenders [18].

Researchers in Uganda measured adherence through pharmacy refills, EAM and TFV‐DP detective devices [18]. Another study, on the other hand, measured high adherence using the consumption of ≥80% of the recommended dosages. The number of returned drugs and reports from SMS showing the number of drug doses taken indicate drug consumption [15].

Acceptability is regarded as the usability and the chances of use (when the efficacy of the rectal gel in stopping HIV transmission is proven) [15]. Participants in both intervention and control groups were reported to have had a positive disposition towards HIV prevention intervention, as the majority (>70%) of enrolees completed all the injection visits. However, cases of participants’ withdrawal from the research were recorded. These were attributable to noncompliance with research procedures, clinical adverse effects of drugs and clinical abnormalities [16]. In another study, participants had a preference for oral medication followed by daily rectal gel and RAI gel [15].

#### Efficacy and tolerability of health intervention

Although the efficacy of the prophylaxis effects of the intervention drug was confirmed, it failed to prevent type 1 HIV acquisition in some of the reviewed articles. However, its efficacy in preventing HIV‐1 infection was only confirmed when the virus was sensitive to the antibody in the administered drug. VRC01 failed to stop the global HIV‐1 transmission, although, in the group where a high dose was administered, the global efficacy in preventing HIV infection was comparatively better than the low‐dose set [17]. The efficacy likewise varied between the two study groups. Furthermore, when intervention drugs are combined, Corey et al. observed that they effectively inhibited the virulency of HIV in populations classified as high‐risk, unlike when a single drug (VRC01 or bnAb) is administered [17]. Moreover, the long‐term drug administration (20 months) offered up to 75% protection against the strain of HIV that was common in the study area, especially at the onset of exposure [17]. As regards tolerability, the intervention drug was well tolerated by all genders across the study sites investigated in one of the articles [16].

#### Health behaviour post‐intervention

Despite the interventions, the non‐use of condoms remained high in one of the reviewed articles [18]. Notwithstanding, some transgenders reported safe practices as indicated by the use of condoms during sexual exploits [18]. Some cases of HIV infection reported by the authors were attributed to participants’ health behaviours. For instance, it was observed that the only positive case of HIV recorded in one of the reviewed articles was due to the non‐use of oral PrEP [18]. Similarly, some cases of HIV infection were also recorded by other authors – five participants contracted HIV in the course of the research [15, 16].

### Confounding factors and intervention outcomes

A study reported some gender differences in the rate of PrEP adherence in their study; they observed that transgenders tend to adhere to PrEP more than other gender groups [18].

### Safety of intervention

Six deaths unrelated to the intervention drugs were recorded. However, some cases of adverse drug effects were observed, ranging from moderate to severe [17]. In another study, there were cases of adverse reactions to the drug, such as injection site reactions, decreased creatinine renal clearance, headaches, influenza and other serious adverse events experienced by some of the participants in the course of the research [16].

## DISCUSSION

The selection criteria for this scoping review identified four eligible papers for analysis. All articles focused on various gender identities and included samples of transgender men and women. However, none of the eligible articles encompassed transabled or transracial individuals. One of the reviewed articles was conducted at a single location, whereas the others were cross‐site studies with participants from Africa, the Americas, South America and Europe. Enrolled participants from Africa were predominantly from Southern, Eastern and South‐Eastern regions, with no coverage of the West, North and Central parts of the continent.

The primary focus of the reviewed articles was on sexual and reproductive health within HIV prevention trials targeting at‐risk populations. Various preventive health interventions were employed, but none of the articles included treatment trials. Across all reviewed articles, common preventive interventions were the use of HIV‐inhibiting medications and HIV screening or testing.

Furthermore, only two articles provided baseline data from their investigations [15, 18]; however, they did not report on whether the health intervention trials affected baseline evidence or addressed issues raised by participants prior to the intervention. Despite this, the interventions led to increased uptake of HIV testing, with notable rates reported. HIV testing is crucial for HIV care and prevention, promoting awareness and mitigating risky behaviours among vulnerable populations [19]. The introduction of self‐testing also increased HIV screening rates, facilitated by the secondary distribution of testing kits by study participants [18].

However, despite the high rates of HIV testing reported in the reviewed articles, participants’ adherence to daily oral PrEP was not influenced significantly. This contradicts findings from a study in South Africa, where HIVST and biofeedback counselling led to increased adherence among postpartum women [20]. Determinants of PrEP adherence, such as age, marital status and occupation [21], were not consistently identified in the reviewed publications. A study in Brazil also reported forgetfulness as a significant barrier to PrEP among the transgender population [22]. The study noted that urban living, stimulant use and perceived barriers were reported as significant obstacles to PrEP adherence, particularly among transgender populations.

Non‐adherence to PrEP negatively impacted the expected protection against HIV infection, with some racial and gender differences observed in PrEP uptake and adherence. African participants were reported to be less inclined towards HIV prevention compared to other backgrounds, whereas transgender individuals showed higher rates of PrEP uptake [17, 18].

The review also indicated that administering intervention medication in familiar forms (e.g. oral or gel) tended to promote drug acceptability and adherence, potentially supporting infection prevention among high‐risk groups. However, the interventions did not significantly affect participants’ behavioural disposition towards HIV prevention, as evidenced by low condom usage despite susceptibility to infection. Some transgender participants adhered to condom use for HIV prevention, but instances of HIV transmission were still reported, attributed partly to noncompliance with oral PrEP use (see also [23, 24]).

The authors claimed the intervention drugs’ efficacy against HIV infection; however, actual prevention was only established with large doses and against specific HIV strains [17, 18]. Combination drug therapies were found to be more effective than single drugs, and prolonged administration offered better protection against prevalent HIV strains. Adverse reactions to medications, including injection site reactions, decreased renal clearance, headaches and influenza‐like symptoms, were noted in the reviewed articles [21].

This review highlights a paucity of scientific publications addressing transgender, transabled and transracial populations in Africa, despite their heightened health risks. This underscores the necessity for more health intervention trials across Africa to inform HIV prevention programmes effectively. The lack of literature on the health problems faced by transgender individuals globally is largely due to their marginalized status and discrimination in many African communities and elsewhere [25, 26, 27, 28, 29].

Furthermore, it raises questions about whether national health systems are adequately equipped to meet the diverse needs of transgender populations, suggesting potential care gaps alongside research gaps [30, 31, 32]. Addressing transgender‐related health disparities requires prioritized research that encompasses broader health issues beyond sexual health, thereby contributing to the development of interventions to mitigate health inequities among transgender populations in Africa and beyond [26].

## CONCLUSION

There are limited studies concerning health intervention trials involving transgender, transabled and transracial individuals in Africa. This paucity of data is a major finding of this review. This scoping review only reviewed four eligible articles. The reviewed studies involved transgender individuals, as there were no eligible articles involving transabled and transracial individuals in Africa, neglecting other health domains. The main limitation of the reviewed articles is the reductionism of sexuality regarding transgender behaviour. All four articles mainly focused on sexual health, HIV preventive drugs and vaccine trials. The articles demonstrated the acceptability of drugs and gels as potential HIV prevention methods. It is commendable that some studies involved transgender individuals despite their marginalization in most African societies. However, more studies are required in different spheres of their health, not only concerning sexual behaviour.

## AUTHOR CONTRIBUTIONS

*Conceptualization; data curation; formal analysis; investigation; methodology; project administration; resources; supervision; validation; visualization; writing – original draft; writing – review and editing*: Jimoh Amzat. *Conceptualization; data curation; funding acquisition; investigation; methodology; project administration; resources; software; supervision; validation; visualization; writing – original draft; writing – review and editing*: Kehinde Kazeem Kanmodi. *Data curation; formal analysis; investigation; methodology; resources; validation; visualization; writing – original draft; writing – review and editing*: Kafayat Aminu. *Data curation; investigation; methodology; resources; writing – original draft*: Abbas Ismail. *Conceptualization; data curation; investigation; methodology; resources; software; writing – original draft*: Afeez Abolarinwa Salami.

## CONFLICT OF INTEREST STATEMENT

The authors have no conflicts of interest to declare.

## FUNDING INFORMATION

This study was self‐funded.

## ETHIC STATEMENT

Being a scoping review, ethical approval is not applicable to this study, as this study did not collect data from human or animal subjects but from an open research repository.

## TRANSPARENCY STATEMENT

The lead author – Jimoh Amzat – affirms that this manuscript is an honest, accurate and transparent account of the study being reported, that no important aspects of the study have been omitted, and that any discrepancies from the study as planned (and, if relevant, registered) have been explained.

## Supporting information

Supporting Information

## Data Availability

Data sharing is not applicable to this article as no new data were created or analysed in this study. All authors have read and approved the final version of the manuscript. Kehinde Kazeem Kanmodi had full access to all of the data in this study and takes complete responsibility for the integrity of the data and the accuracy of the data analysis.
