# Supplementary material for: Health intervention trials involving transgender, transabled and transracial persons in Africa: A scoping review
Source: Public Health Chall. 2024 May 6;3(2):e182. doi: 10.1002/puh2.182 (PMC12039567; doi:10.1002/puh2.182)
Supplement: Supplementary file 1 — Supporting Information [file PUH2-3-e182-s001.docx]

**SUPPLEMENTARY FILE**

**Table S1. Search string for PubMed database search**

| **Tag** | **Subject search** | **Search String** |
| --- | --- | --- |
| #1 | People who identify as transgender, transracial or transabled | ((Transgender[Title/Abstract]) OR (transabled[Title/Abstract])) OR (transracial[Title/Abstract]) |
| #2 | Intervention | ((((intervention[Title/Abstract]) OR (program*[Title/Abstract])) OR (trial[Title/Abstract])) OR (experiment*[Title/Abstract])) OR (quasi-experiment*[Title/Abstract]) |
| #3 | African countries, dependencies, and territories | (((((((((((((((((((((((((((((((((((((((((((((((((((((((((((Algeria[MeSH Terms]) OR (Angola[MeSH Terms])) OR (Benin[MeSH Terms])) OR (Botswana[MeSH Terms])) OR (burkina faso[MeSH Terms])) OR (burundi[MeSH Terms])) OR (cabo verde[MeSH Terms])) OR (cape verde[MeSH Terms])) OR (cameroon[MeSH Terms])) OR (central african republic[MeSH Terms])) OR (chad[MeSH Terms])) OR (comoros[MeSH Terms])) OR (congo[MeSH Terms])) OR (ivory coast[MeSH Terms])) OR (cote d ivoire[MeSH Terms])) OR (djibouti[MeSH Terms])) OR (democratic republic of congo[MeSH Terms])) OR (egypt[MeSH Terms])) OR (equatorial guinea[MeSH Terms])) OR (eritrea[MeSH Terms])) OR (eswatini[MeSH Terms])) OR (ethiopia[MeSH Terms])) OR (gabon[MeSH Terms])) OR (gambia[MeSH Terms])) OR (ghana[MeSH Terms])) OR (guinea[MeSH Terms])) OR (guinea bissau[MeSH Terms])) OR (kenya[MeSH Terms])) OR (lesotho[MeSH Terms])) OR (liberia[MeSH Terms])) OR (libya[MeSH Terms])) OR (madagascar[MeSH Terms])) OR (malawi[MeSH Terms])) OR (mali[MeSH Terms])) OR (mauritania[MeSH Terms])) OR (mauritius[MeSH Terms])) OR (morocco[MeSH Terms])) OR (mozambique[MeSH Terms])) OR (namibia[MeSH Terms])) OR (niger[MeSH Terms])) OR (nigeria[MeSH Terms])) OR (rwanda[MeSH Terms])) OR (sao tome and principe[MeSH Terms])) OR (senegal[MeSH Terms])) OR (seychelles[MeSH Terms])) OR (sierra leone[MeSH Terms])) OR (somalia[MeSH Terms])) OR (south africa[MeSH Terms])) OR (south sudan[MeSH Terms])) OR (sudan[MeSH Terms])) OR (tanzania[MeSH Terms])) OR (togo[MeSH Terms])) OR (tunisia[MeSH Terms])) OR (uganda[MeSH Terms])) OR (zambia[MeSH Terms])) OR (zimbabwe[MeSH Terms])) OR (reunion[MeSH Terms])) OR (saint helena[MeSH Terms])) OR (western sahara[MeSH Terms])) OR (mayotte[MeSH Terms]) |
| #3 | #1 AND #2 AND #3 | (#1) AND (#2) AND (#3) |

**Table S2. Search string for SCOPUS database search**

| **Tag** | **Subject Search** | **Search String** |
| --- | --- | --- |
| #1 | People who identify as transgender, transracial or transabled | ( TITLE-ABS-KEY ( transgender )  OR  TITLE-ABS-KEY ( transabled )  OR  TITLE-ABS-KEY ( transracial ) ) |
| #2 | Intervention | ((((intervention[Title/Abstract]) OR (program*[Title/Abstract])) OR (trial[Title/Abstract])) OR (experiment*[Title/Abstract])) OR (quasi-experiment*[Title/Abstract]) |
| #3 | African countries, dependencies, and territories | ( ( TITLE-ABS-KEY ( angola )  OR  TITLE-ABS-KEY ( benin )  OR  TITLE-ABS-KEY ( botswana )  OR  TITLE-ABS-KEY ( burkina  AND  faso )  OR  TITLE-ABS-KEY ( burundi )  OR  TITLE-ABS-KEY ( cameroon )  OR  TITLE-ABS-KEY ( cabo  AND  verde )  OR  TITLE-ABS-KEY ( cape  AND  verde )  OR  TITLE-ABS-KEY ( central  AND  african  AND  republic )  OR  TITLE-ABS-KEY ( chad )  OR  TITLE-ABS-KEY ( comoros )  OR  TITLE-ABS-KEY ( congo )  OR  TITLE-ABS-KEY ( ivory  AND  coast )  OR  TITLE-ABS-KEY ( democratic  AND  republic  AND  of  AND  congo )  OR  TITLE-ABS-KEY ( djibouti )  OR  TITLE-ABS-KEY ( equatorial  AND  guinea )  OR  TITLE-ABS-KEY ( eritrea )  OR  TITLE-ABS-KEY ( ethiopia )  OR  TITLE-ABS-KEY ( gabon )  OR  TITLE-ABS-KEY ( gambia )  OR  TITLE-ABS-KEY ( ghana )  OR  TITLE-ABS-KEY ( guinea )  OR  TITLE-ABS-KEY ( guinea-bissau )  OR  TITLE-ABS-KEY ( kenya )  OR  TITLE-ABS-KEY ( lesotho )  OR  TITLE-ABS-KEY ( liberia )  OR  TITLE-ABS-KEY ( madagascar )  OR  TITLE-ABS-KEY ( malawi )  OR  TITLE-ABS-KEY ( mali )  OR  TITLE-ABS-KEY ( mauritania )  OR  TITLE-ABS-KEY ( mauritius )  OR  TITLE-ABS-KEY ( mayotte )  OR  TITLE-ABS-KEY ( mozambique )  OR  TITLE-ABS-KEY ( namibia )  OR  TITLE-ABS-KEY ( niger )  OR  TITLE-ABS-KEY ( nigeria )  OR  TITLE-ABS-KEY ( reunion )  OR  TITLE-ABS-KEY ( rwanda )  OR  TITLE-ABS-KEY ( saint  AND  helena )  OR  TITLE-ABS-KEY ( sao  AND  tome  AND  principe )  OR  TITLE-ABS-KEY ( senegal )  OR  TITLE-ABS-KEY ( seychelles )  OR  TITLE-ABS-KEY ( sierra  AND  leone )  OR  TITLE-ABS-KEY ( somalia )  OR  TITLE-ABS-KEY ( south  AND  africa )  OR  TITLE-ABS-KEY ( south  AND  sudan ) ) )  OR  ( ( TITLE-ABS-KEY ( eswatini )  OR  TITLE-ABS-KEY ( togo )  OR  TITLE-ABS-KEY ( uganda )  OR  TITLE-ABS-KEY ( zambia )  OR  TITLE-ABS-KEY ( zimbabwe )  OR  TITLE-ABS-KEY ( egypt )  OR  TITLE-ABS-KEY ( libya )  OR  TITLE-ABS-KEY ( algeria )  OR  TITLE-ABS-KEY ( tunisia )  OR  TITLE-ABS-KEY ( morocco )  OR  TITLE-ABS-KEY ( western  AND  sahara )  OR  TITLE-ABS-KEY ( sudan )  OR  TITLE-ABS-KEY ( tunisia ) ) ) |
| #4 | #1 AND #2 AND #3 | (#1) AND (#2) AND (#3) |

**Table S3. Search string for other databases (AMED – The Allied and Complementary Medicine Database; APA PsycInfo and CINAHL Complete) search via EBSCO interface**

| **Tag** | **Subject Search** | **Search String** |
| --- | --- | --- |
| S1 | People who identify as transgender, transracial or transabled | AB transgender OR AB transabled OR AB transracial |
| S2 | Intervention | AB intervention OR AB program* OR AB trial OR AB experiment* OR AB quasi-experiment* |
| S3 | African countries, dependencies, and territories | AB algeria OR AB angola OR AB benin OR AB botswana OR AB burkina faso OR AB burundi OR AB cape verde OR AB cabo verde OR AB cameroon OR AB central african republic OR AB chad OR AB comoros OR AB congo OR AB cote d'ivoire OR AB ivory coast OR AB djibouti OR AB democratic republic of congo OR AB egypt OR AB equatorial guinea OR AB eritrea OR AB eswatini OR AB ethiopia OR AB gabon OR AB gambia OR AB ghana OR AB guinea OR AB guinea bissau OR AB kenya OR AB lesotho OR AB liberia OR AB libya OR AB madagascar OR AB malawi OR AB mali OR AB mauritania OR AB mauritius OR AB morocco OR AB mozambique OR AB namibia OR AB niger OR AB nigeria OR AB rwanda OR AB ( sao tome and principe ) OR AB senegal OR AB seychelles OR AB sierra leone OR AB somalia OR AB south Africa OR AB south sudan OR AB sudan OR AB tanzania OR AB togo OR AB tunisia OR AB uganda OR AB zambia OR AB zimbabwe OR AB reunion OR AB saint helena OR AB western sahara OR AB mayotte |
| S4 | S1 AND S2 AND S3 | S1 AND S2 AND S3 |

**Table S4. List of literatures whose full texts were screened for inclusion/exclusion into the scoping review**

| **No.** | **Citations** | **Included** | **Excluded (Reasons)** |
| --- | --- | --- | --- |
| 1 | Mwango, L., Toeque, M. G., Lindsay, B., Tembo, K., Sakala, H., Reggee, S., Malunga, S. M., Kabwe, M., Kafunda, I., Olufunso, A., Mwila, A., Okuku, J., Kancheya, N., Nkwemu, K., Mumba, D., Hachaambwa, L., Sheneberger, R., Blanco, N., Lavoie, M. C., Stafford, K. A., … Claassen, C. W. (2022). Reaching transgender populations in Zambia for HIV prevention and linkage to treatment using community-based service delivery. *Journal of the International AIDS Society*, *25 Suppl 5*(Suppl 5), e25995. https://doi.org/10.1002/jia2.25995 |  | Yes (Wrong study design) |
| 2 | Kimani, M., Sanders, E. J., Chirro, O., Mukuria, N., Mahmoud, S., Rinke de Wit, T. F., Graham, S. M., Operario, D., & van der Elst, E. M. (2022). Pre-exposure prophylaxis for transgender women and men who have sex with men: qualitative insights from healthcare providers, community organization-based leadership and end users in coastal Kenya. *International health*, *14*(3), 288–294. https://doi.org/10.1093/inthealth/ihab043 |  | Yes (Wrong study design) |
| 3 | Mujugira, A., Nakyanzi, A., Nabaggala, M. S., Muwonge, T. R., Ssebuliba, T., Bagaya, M., Nampewo, O., Sapiri, O., Nyanzi, K. R., Bambia, F., Nsubuga, R., Serwadda, D. M., Ware, N. C., Baeten, J. M., & Haberer, J. E. (2022). Effect of HIV Self-Testing on PrEP Adherence Among Gender-Diverse Sex Workers in Uganda: A Randomized Trial. *Journal of acquired immune deficiency syndromes (1999)*, *89*(4), 381–389. https://doi.org/10.1097/QAI.0000000000002895 | Yes |  |
| 4 | Sullivan, P. S., Phaswana-Mafuya, N., Baral, S. D., Valencia, R., Zahn, R., Dominguez, K., Yah, C. S., Jones, J., Kgatitswe, L. B., McNaghten, A. D., Siegler, A. J., Sanchez, T. H., & Bekker, L. G. (2020). HIV prevalence and incidence in a cohort of South African men and transgender women who have sex with men: the Sibanye Methods for Prevention Packages Programme (MP3) project. *Journal of the International AIDS Society*, *23 Suppl 6*(Suppl 6), e25591. https://doi.org/10.1002/jia2.25591 |  | Yes (Wrong study design) |
| 5 | Buchbinder, S. P., Glidden, D. V., Liu, A. Y., McMahan, V., Guanira, J. V., Mayer, K. H., Goicochea, P., & Grant, R. M. (2014). HIV pre-exposure prophylaxis in men who have sex with men and transgender women: a secondary analysis of a phase 3 randomised controlled efficacy trial. *The Lancet. Infectious diseases*, *14*(6), 468–475. https://doi.org/10.1016/S1473-3099(14)70025-8 |  | Yes (Wrong study design) |
| 6 | Corey, L., Gilbert, P. B., Juraska, M., Montefiori, D. C., Morris, L., Karuna, S. T., Edupuganti, S., Mgodi, N. M., deCamp, A. C., Rudnicki, E., Huang, Y., Gonzales, P., Cabello, R., Orrell, C., Lama, J. R., Laher, F., Lazarus, E. M., Sanchez, J., Frank, I., Hinojosa, J., … HVTN 704/HPTN 085 and HVTN 703/HPTN 081 Study Teams (2021). Two Randomized Trials of Neutralizing Antibodies to Prevent HIV-1 Acquisition. *The New England journal of medicine*, *384*(11), 1003–1014. https://doi.org/10.1056/NEJMoa2031738 | Yes |  |
| 7 | Liu, A. Y., Norwood, A., Gundacker, H., Carballo-Diéguez, A., Johnson, S., Patterson, K., Bekker, L. G., Chariyalertsak, S., Chitwarakorn, A., Gonzales, P., Holtz, T. H., Mayer, K. H., Zorrilla, C., Buchbinder, S., Piper, J. M., Lama, J. R., & Cranston, R. D. (2019). Brief Report: Routine Use of Oral PrEP in a Phase 2 Rectal Microbicide Study of Tenofovir Reduced-Glycerin 1% Gel (MTN-017). *Journal of acquired immune deficiency syndromes (1999)*, *81*(5), 516–520. https://doi.org/10.1097/QAI.0000000000002066 |  | Yes (Wrong study design) |
| 8 | Landovitz, R. J., Li, S., Grinsztejn, B., Dawood, H., Liu, A. Y., Magnus, M., Hosseinipour, M. C., Panchia, R., Cottle, L., Chau, G., Richardson, P., Marzinke, M. A., Hendrix, C. W., Eshleman, S. H., Zhang, Y., Tolley, E., Sugarman, J., Kofron, R., Adeyeye, A., Burns, D., … Eron, J. J. (2018). Safety, tolerability, and pharmacokinetics of long-acting injectable cabotegravir in low-risk HIV-uninfected individuals: HPTN 077, a phase 2a randomized controlled trial. *PLoS medicine*, *15*(11), e1002690. https://doi.org/10.1371/journal.pmed.1002690 | Yes |  |
| 9 | Eakle, R., Bourne, A., Mbogua, J., Mutanha, N., & Rees, H. (2018). Exploring acceptability of oral PrEP prior to implementation among female sex workers in South Africa. *Journal of the International AIDS Society*, *21*(2), e25081. https://doi.org/10.1002/jia2.25081 |  | Yes (Wrong study design) |
| 10 | Cranston, R. D., Lama, J. R., Richardson, B. A., Carballo-Diéguez, A., Kunjara Na Ayudhya, R. P., Liu, K., Patterson, K. B., Leu, C. S., Galaska, B., Jacobson, C. E., Parikh, U. M., Marzinke, M. A., Hendrix, C. W., Johnson, S., Piper, J. M., Grossman, C., Ho, K. S., Lucas, J., Pickett, J., Bekker, L. G., … MTN-017 Protocol Team (2017). MTN-017: A Rectal Phase 2 Extended Safety and Acceptability Study of Tenofovir Reduced-Glycerin 1% Gel. *Clinical infectious diseases : an official publication of the Infectious Diseases Society of America*, *64*(5), 614–620. https://doi.org/10.1093/cid/ciw832 | Yes |  |
| 11 | Gandhi, M., Glidden, D. V., Mayer, K., Schechter, M., Buchbinder, S., Grinsztejn, B., Hosek, S., Casapia, M., Guanira, J., Bekker, L. G., Louie, A., Horng, H., Benet, L. Z., Liu, A., & Grant, R. M. (2016). Association of age, baseline kidney function, and medication exposure with declines in creatinine clearance on pre-exposure prophylaxis: an observational cohort study. *The lancet. HIV*, *3*(11), e521–e528. https://doi.org/10.1016/S2352-3018(16)30153-9 |  | Yes (Wrong study design) |
| 12 | London, S. (2014). No Increase in Sexual Risk Behaviors Seen After Men at Risk of HIV Start Preexposure Prophylaxis. *Perspectives on Sexual and Reproductive Health*, *46*(1), 51-52. |  | Yes (Wrong publication type) |
